# An Ultrasonic Reverse Time Migration Imaging Method Based on Higher-Order Singular Value Decomposition

**DOI:** 10.3390/s22072534

**Published:** 2022-03-25

**Authors:** Yuncheng Zhang, Xiang Gao, Jiawei Zhang, Jingpin Jiao

**Affiliations:** Faculty of Materials and Manufacturing, Beijing University of Technology, Beijing 100124, China; zhangyuncheng@emails.bjut.edu.cn (Y.Z.); zhangmeilan@emails.bjut.edu.cn (J.Z.); jiaojp@bjut.edu.cn (J.J.)

**Keywords:** reverse time migration, higher-order singular value decomposition, ultrasonic nondestructive testing, coarse-grained material, noise reduction

## Abstract

An ultrasonic reverse time migration imaging method, based on high-order singular value decomposition, is proposed in the study to solve the problems of low signal-to-noise ratio (SNR) and excessive artifacts in defect ultrasonic detection imaging results of materials with high noise levels. In this method, based on the 3D structural properties of the ultrasonic full-matrix capture data, higher-order singular value decomposition is directly performed with the 3D data. The method overcomes the difficulty in selecting the number of singular values in the original singular value decomposition noise-reduction algorithm and realizes the one-step noise reduction processing of all the signals. Subsequently, the reverse time migration imaging is performed in the frequency domain, and high-quality acoustic images are obtained. The effects of the number of array elements, the center frequency of the excitation signal, and the number of defects on the denoising effect of the algorithm are investigated. It was experimentally demonstrated that the method could suppress the interference of noise signals and significantly improve the imaging SNR compared with total focusing method and the reverse time migration.

## 1. Introduction

Ultrasonic testing is one of the most commonly used nondestructive testing and evaluation (NDT&E) techniques. Compared to single-element transducers, the arrays perform well in terms of inspection performance, flexibility, and imaging speed [1]. Due to the rapid development of array ultrasound, a variety of imaging algorithms have been developed [2]. However, most of the ultrasonic imaging algorithms are not applicable to the materials with high noise levels, such as high-density polyethylene (HDPE) pipes, coarse-grained austenitic–ferritic steels, and steel ingots. The highly heterogeneous structures of these materials produce a strong coherent scattering noise, so that the signal amplitude is reduced to a level close or even lower than the noise level [3]. The ultrasonic imaging technique presents a low signal-to-noise ratio (SNR) in these materials [4]. Therefore, it is necessary to develop an ultrasonic imaging method for the materials with high noise levels.

The received signals in ultrasonic NDE are commonly contaminated by noise originating from both measurement systems and measured samples. The noise originating from a measurement system can be eliminated by signal averaging, digital filtering, and other methods because its distribution characteristics are different from defective echo signal in the time domain and frequency domain [5]. Noise originating from a measured sample is also called structural noise, which refers to the acoustic noise caused by the scattering of acoustic waves due to the structural properties of the material. The structural noise in metallic materials is mostly caused by the scattering of ultrasonic waves at grain boundaries [6]. Structural noise is also correlated with defect echoes, so the elimination of structural noise may also lead to the loss of useful defect echoes [7]. Structural noise affects defect detection accuracy and imaging quality. Therefore, it is necessary to eliminate structural noise in the field of ultrasonic inspection.

Commonly used denoising methods of structural noise include split-spectrum processing (SSP) technique [8], wavelet transformation method (WTM), and singular value decomposition (SVD) [9]. In split-spectrum processing (SSP), a set of narrow-band filters are used to separate and reconstruct the ultrasonic echo signal according to the different sensitivity of the noise signal and the defective signal to frequency changes [10]. Although this method reduces noise significantly, its denoising performance is excessively dependent on filter selection, center frequency, and bandwidth, thus limiting its application in ultrasonic signal noise reduction [11]. Praveen et al. [12] used high-order wavelets to analyze its denoising performance in processing the ultrasonic signals of austenitic stainless-steel welds and verified the performance of high-order wavelet analysis. Wei et al. [13] proposed a wavelet packet noise-reduction method with an adjustable threshold value, thus breaking the limitations of soft and hard thresholding methods. WTM has a certain denoising ability for ultrasonic signals, but it requires the careful and difficult selection of wavelet basis functions and a complex processing step to obtain wavelet thresholds and threshold functions. Another widely studied class of noise reduction methods are based on singular value decomposition or eigenvalue decomposition. Saniie et al. [14,15] performed SVD with time–frequency matrix of data and proved that the SVD method still had the good noise reduction performance in processing low SNR signals. However, this method might neglect some useful signals in the same frequency band. Prada et al. [16] described a technique based on decomposition of the time-reversal operator (DORT), which integrated a time-reversal operator (TRO) with SVD. The method was successfully applied to analyze a variety of materials with high noise levels, indicating that DOTR could significantly reduce the structural noise in the signal and retain the useful signal that overlapped the scattered noise signal band [17,18]. The key to the noise-reduction method is to determine the number of singular values of useful signal and noise signal, but a determination method of the number of singular values is not available [19]. Higher-order singular value decomposition (HOSVD), known as Tucker decomposition, is a multilinear extension of the concept of SVD and is commonly used in tensor decomposition [20]. HOSVD has been extensively studied in fault diagnosis, computer vision, radar detection, and other fields [21,22]. Hu C. and Wang Y. used the rolling bearing vibration signal to construct a three-dimensional tensor for truncate HOSVD and realized the fault detection of rolling bearings after denoising signals [23]. Some scholars stacked similar image blocks into three-dimensional arrays and performed hard threshold coefficient shrinkage with the core tensor obtained by HOSVD to achieve image noise reduction, while preserving the two-dimensional structure and features of image blocks [24]. Some scholars developed a radio frequency interference mitigation method based on the HOSVD algorithm and orthogonal subspace projection and the method showed significant advantages in terms of retaining the desired signals while suppressing interference [25]. These studies showed that HOSVD method had unique advantages in data processing and could achieve the elimination of high-dimensional signal noise while preserving original signal characteristics.

In most of the above noise-reduction algorithms, total focusing method (TFM) is used to create detection images [26]. TFM is an imaging method based on full-matrix capture (FMC) data [27]. Reverse time migration (RTM) has been extensively studied in geophysical exploration and other fields [28,29] and is an imaging method based on FMC data. In recent years, this method has been applied in the field of ultrasonic testing and promising results have been achieved [30]. Compared with TFM, RTM is more advantageous in defect detection of irregular shapes [31]. Anderson et al. [32] applied RTM imaging in bounded samples, where reverberation always existed. This method realized defect detection in plate structures and the compensation for guided wave dispersion effects [33,34]. Compared with the traditional ultrasonic imaging method, the RTM method can reconstruct the images of buried sleeves with a higher resolution and a greatly improved accuracy [35]. Gao X et al. [36] successfully detected defects in multilayer media with RTM method. In general, reverse time migration imaging is an effective imaging tool in the field of ultrasonic imaging, but this method has been seldom applied in the defect detection of the materials with high noise levels.

In this study, an ultrasonic reverse time migration imaging method based on higher-order singular value decomposition is proposed. This method overcomes the difficulty in determining the number of singular values in the original SVD algorithm. In this method, three-dimensional data are directly processed to denoise all signals through a single step. In the subsequent imaging process, the RTM imaging technique is applied in the frequency domain to reduce the computation time and improve the imaging quality. The proposed method allows the high-quality ultrasonic imaging of defects in the materials with high noise levels. This method is applicable to ultrasonic inspection, medical imaging, and geophysical exploration.

## 2. Materials and Methods

### 2.1. Working Principle

**Figure 1:**
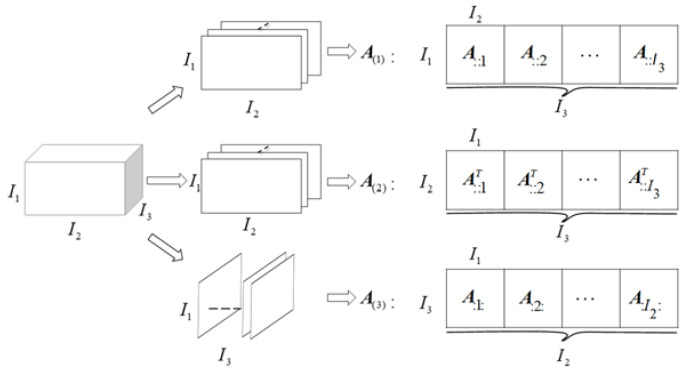
Unfolding of the third-order tensor.

A higher-order tensor can be represented as $A\in {\mathbb{C}}^{I_{1}\times I_{2}\times I_{3}\ldots \times I_{n}}$, where *n* is the order of the tensor. The third-order tensor is the most commonly used tensor, and Figure 1 is a schematic diagram of the unfolding of the third-order tensor model. The mode-*n* unfolding (also called matricization or flattening) of the third-order tensor $A\in {\mathbb{C}}^{I_{1}\times I_{2}\times I_{3}}$ is defined as the unfolding process of a three-dimensional array into a two-dimensional matrix $A_{\left(n\right)}$. According to the Kolda method [37,38], the third-order tensor elements are mapped into the elements $\left(i_{n},j\right)$ of the mode-*n* matrix $A_{\left(n\right)}$:

(1) $$j=1+\overset{3}{\underset{k=1,k\;\neq \;n}{\sum }}\left[\left(i_{k}-1\right)\overset{k-1}{\underset{m=1,m\;\neq \;n}{\prod }}I_{m}\right]\;\;\;n\;=1,2,3,$$

In order to mine the information in tensor data, the tensor needs to be decomposed. The higher-order singular value decomposition of the third-order tensor $A\in {\mathbb{C}}^{I_{1}\times I_{2}\times I_{3}}$ is expressed as follows:

(2) $$A=S\times_{1}U_{\left(1\right)}\times_{2}U_{\left(2\right)}\times_{3}U_{\left(3\right)},$$

where the symbol $\times_{n}$ denotes the *n*-mode product; $S\in {\mathbb{C}}^{I_{1}\times I_{2}\times I_{3}}$ is called the core tensor, and can be obtained as follows:

(3) $$S=A\times_{1}U_{\left(1\right)}^{T}\times_{2}U_{\left(2\right)}^{T}\times_{3}U_{\left(3\right)}^{T},$$

$U_{\left(1\right)}$, $U_{\left(2\right)}$, and $U_{\left(3\right)}$ are the standard orthogonal column vector spaces that can be obtained from the singular value decomposition of the mode-*n* matrix $A_{\left(n\right)}$, as follows:

(4) $$A_{\left(n\right)}=U_{\left(n\right)}\sum_{\left(n\right)}V_{\left(n\right)}\;\;\;n=1,\;2,\;3,$$

Through the singular value decomposition, the matrix $A_{\left(n\right)}$ is decomposed into the product of three matrices. $\Sigma_{\left(n\right)}=diag\left(\sigma_{n,1},\ldots ,\sigma_{n,I_{n}}\right)$ is the most important singular value matrix. $\sigma_{n,k}$ is the *k*-th singular value of $\Sigma_{\left(n\right)}$ and indicates the importance of the *k*-th column vector $u_{n,k}$ in the left singular matrix $U_{\left(n\right)}$.

In ultrasonic phased array inspection, the full-matrix capture (FMC) data obtained by the phased array can be represented as a third-order tensor $A\in {\mathbb{C}}^{N\times N\times M}$, where *N* is the number of array elements and *M* is the signal length. The tensor $A\in {\mathbb{C}}^{N\times N\times M}$ is unfolded in the frequency domain after Fourier transformation to obtain the matrix $A_{\left(1\right)}$. The matrix $A_{\left(1\right)}$ represents a regular arrangement of the values received by each array element at all frequencies and contains all information of the FMC data. Therefore, the matrix $A_{\left(1\right)}$ can be used in defect detection.

The reverse time migration (RTM) algorithm is a new defect imaging method that consists of three primary steps: forward propagation of the source wavefield, reverse propagation of the received wavefield, and application of imaging conditions. The cross-correlation imaging condition between the forward-propagated source wavefield and backward-propagated receiver wavefield is usually used. RTM in the frequency domain is expressed as follows:

(5) $$I\left(x,y\right)=abs\left[\overset{N}{\underset{i=1}{\sum }}\overset{N}{\underset{j=1}{\sum }}\underset{\omega }{\sum }S\left(\omega \right)R^{*}\left(\omega \right)e^{-jk\left(r_{1}+r_{2}\right)}\right],$$

where ω is the angular frequency; k is the wave number; I(x,y) denotes the image value for the grid point (x,y); $r_{1}$ is the distance from the grid point to the excitation array element; $r_{2}$ is the distance from the grid point to the receiving array element; the symbol * represents the conjugate of the matrix; and R(ω) are the components of the forward-propagated source wavefield and backward-propagated receiver wavefield at a certain frequency.

The source wavefield is expressed as a tensor $ℬ\in {\mathbb{C}}^{N\times N\times M}$ of N×N channels, each of which is the same frequency domain excitation signal. Equation (5) can be rewritten as:

(6) $$I\left(x,y\right)=abs\left[\overset{N}{\underset{i=1}{\sum }}\overset{MN}{\underset{j=1}{\sum }}C\left(i,j\right)\right],$$

(7) $$C=A_{\left(1\right)}^{*}\circ B_{\left(1\right)}\circ E,$$

where $C\in {\mathbb{C}}^{N\times MN}$; the symbol ∘ is the Hadamard product of the matrix; $A_{\left(1\right)}^{*}$ is the conjugate matrix of $A_{\left(1\right)}$; $A_{\left(1\right)}$ is the mode-1 matrix of the received wavefield; $B_{\left(1\right)}$ is the mode-1 matrix of the source wavefield; E is a matrix with the same dimensions of $A_{\left(1\right)}$ and $B_{\left(1\right)}$ its elements are expressed as follows:

(8) $$E\left(i,j\right)=e^{-jk_{n}r_{\left(x,y\right)}},$$

where $k_{n}$ is the wave number corresponding to A(i,j); $r_{\left(x,y\right)}$ is the sum of the distances from the point (x,y) to corresponding excitation array element and corresponding reception array element.

When A is contaminated by noise, the elements in with smaller singular values are mainly caused by noise. Since the magnitude of the singular values indicates the importance of their corresponding singular vectors in the data, we can define weight coefficients in terms of all singular values in $\Sigma_{\left(1\right)}$ to attenuate the effect of the noise vector subspace. The weighting coefficient $a_{1}\left(i\right)$ is defined as follows:

(9) $$a_{1}\left(i\right)=\left[\frac{\sigma^{1}}{\sigma^{N}},\frac{\sigma^{2}}{\sigma^{N}},\ldots \frac{\sigma^{i}}{\sigma^{N}}\ldots ,\frac{\sigma^{N}}{\sigma^{N}}\right],$$

After multiplying $a_{1}\left(i\right)$ by the corresponding column vector in $U_{\left(1\right)}$, the new left singular matrix $U_{\left(1\right)}{}^{new}$ is obtained as follows:

(10) $$U_{\left(1\right)}{}^{new}=\left[u_{1}*a\left(1\right),u_{2}*a\left(2\right),\ldots ,u_{i}*a\left(i\right),\ldots ,u_{N}*a\left(N\right)\right],$$

**Figure 2:**
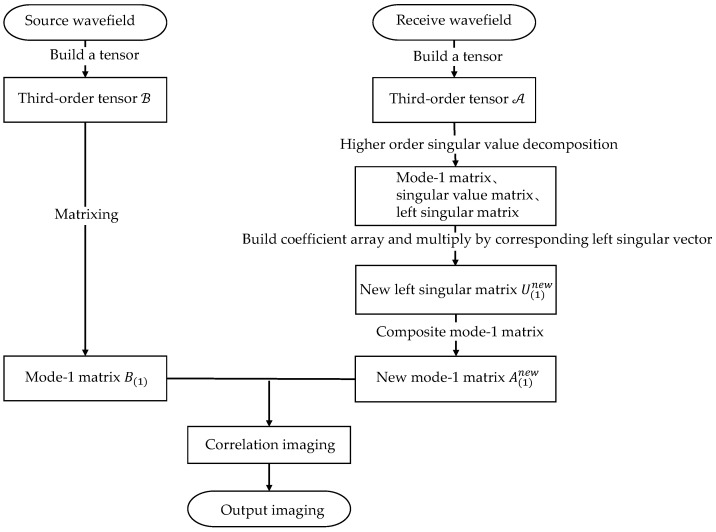
Unfolding of the third-order tensor.

Substituting $U_{\left(1\right)}{}^{new}$ into Equation (4) yields the new mode-1 matrix $A_{\left(1\right)}{}^{new}$ and the combination of Equations (6) and (7) yields the following noise-reduced imaging equation:

(11) $$A_{\left(1\right)}^{new}=U_{\left(1\right)}^{new}\sum_{\left(1\right)}V_{\left(1\right)},$$

(12) $$C^{new}=A_{\left(1\right)}^{new}{}^{*}\circ B_{\left(1\right)}\circ E,$$

(13) $$I^{new}\left(x,y\right)=abs\left[\overset{N}{\underset{i=1}{\sum }}\overset{MN}{\underset{j=1}{\sum }}C\left(i,j\right)\right]$$

where $I^{new}\left(x,y\right)$ denotes the final image value for the grid point. In this way, the noise interference is removed while completing the imaging. The full-matrix frequency domain data tensor after noise suppression can be obtained by substituting $A_{\left(1\right)}{}^{new}$ into Equation (2) and the noise reduction effect of this algorithm can be demonstrated in the time domain after Fourier transformation. The higher-order singular value decomposition-based reverse time migration (HOSVD-RTM) algorithm is shown in Figure 2.

### 2.2. Numerical Modeling Setup

**Figure 3:**
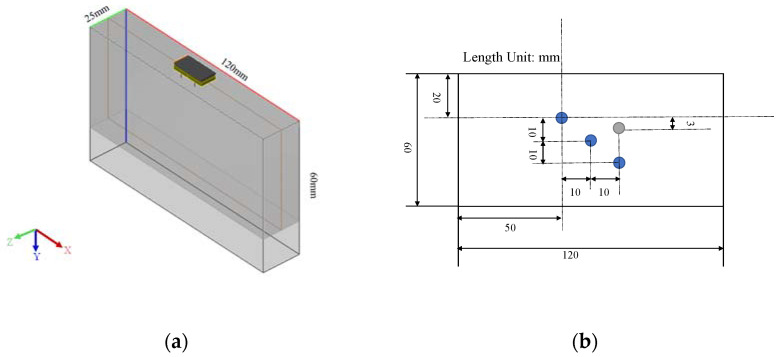
(**a**) Simulation model; (**b**) schematic diagram.

In this study, CIVA software is used for numerical simulation. The simulation model and corresponding schematic diagram are shown in Figure 3. The model size is 120 × 60 × 25 mm^3^. The material is set to 45# steel, and its density is 7.8 $\mathrm{kg}/m^{3}$. In this simulation model, we only calculated the propagation of longitudinal waves, and the longitudinal wave speed is 5820 m/s. The linear ultrasound array is chosen for the simulation. The center of the linear array is located at the center of the upper surface of the tested block. The data acquisition method is full-matrix capture. The direct wave or bottom echo signals are not set in the simulation, so the obtained scattered signal contains only the features of defects.

### 2.3. Experiment Setup

**Figure 4:**
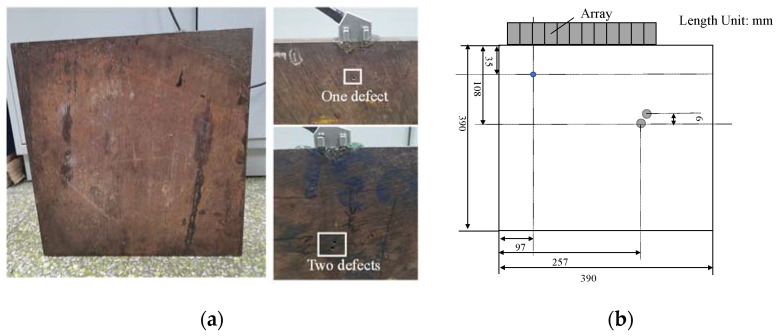
(**a**) Physical diagram; (**b**) schematic diagram of the experimental specimen.

In this study, a Multi2000 ultrasonic phased-array inspection system was used for the experimental verification. The system mainly consisted of a computer, a Multi2000 phased array detector and ultrasound phased arrays. In order to test the applicability of the proposed algorithm in the materials with high noise levels, 4140 casting steel was selected as the tested material. The internal defect detection of this material is particularly crucial since it is used in the manufacture of booster transmission gears, connecting rods bearing large loads, and structural components such as spring clips. The material’s chemical composition is as follows (in weight%): C 0.38–0.45, Si 0.17–0.37, Mn 0.60–0.80, Cr 0.9–1.2, Mo 0.15–0.25, S ≤ 0.07, *p* ≤ 0.07, Al ≤ 0.25, and Fe(balance). The material has a large internal grain size and more grain structures, thus resulting in the more structural noise in acquired signals. The size of the experimental specimen is 390 × 390 × 55 mm^3^ and the physical diagram and schematic diagram of the experimental specimen are shown in Figure 4.

## 3. Results and Discussion

### 3.1. Numerical Simulation Results

#### 3.1.1. A Single Defect

A single circular hole defect is introduced into the simulation model for inspection, as indicated by the gray circle in Figure 3b. The top left vertex in Figure 3b is defined as the origin, so that the center of the defect is located at the point (70 mm, 23 mm). The diameter of the circular hole is 2 mm. The 32 elements on the top surface of the linear array are spaced at a fixed pitch of 0.6 mm between their centers. In this section, the 5-cycle Hanning window pulse with a center frequency of 5 MHz is selected as the excitation signal. Gaussian noise is added to the acquired simulation data to simulate the scattering noise in actual detection. The standard deviation σ of the Gaussian noise added in this section is half of the peak value of the defect echo.

**Figure 5:**
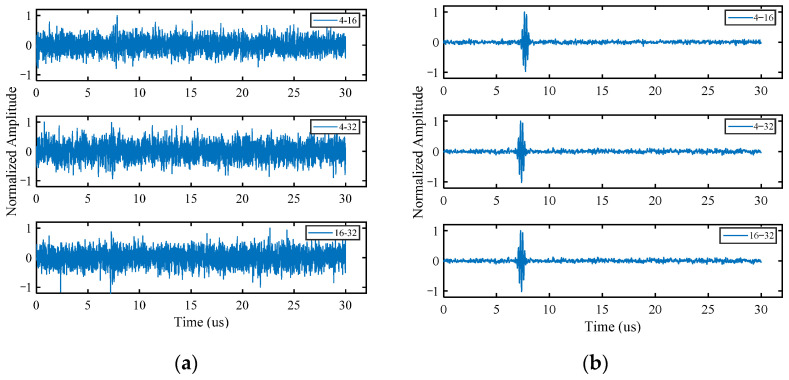
Time domain waveforms of simulated signals. (**a**) Before noise reduction; (**b**) after noise reduction.

Following Fourier transformation, the received FMC data are decomposed in the frequency domain with higher-order singular values and the noise-suppressed mode-1 matrix $A_{\left(1\right)}{}^{new}$ is obtained with Equation (11). For the purpose of the more intuitive display of the denoising results, $A_{\left(1\right)}{}^{new}$ is substituted into Equation (2) to obtain a three-dimensional frequency domain matrix, which is then subjected to the inverse Fourier transformation for obtaining the time domain waveform. Figure 5 shows the comparison of the normalized time domain waveforms before and after noise reduction for different transmission–reception pairs.

The noise signal is significantly reduced by the noise reduction method of HOSVD, and the echo signal of defects is highlighted (Figure 5). The algorithm is still effective even when the defect signal is almost completely overlapped by the noise signal. The signals of all transmission–reception pairs obtain similar noise reduction effect simultaneously, so that it is not necessary to process the data of each channel separately. Therefore, this method can improve the computational efficiency and is conducive to processing multi-array signal data.

**Figure 6:**
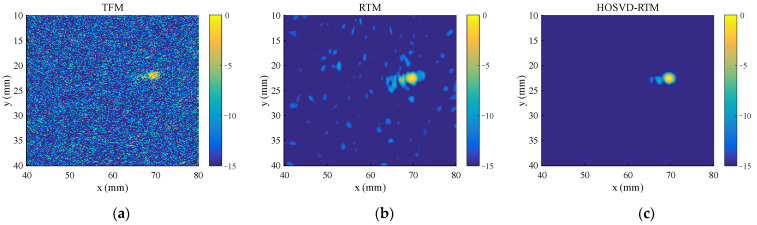
Simulated signal imaging results obtained with various algorithms. (**a**) TFM; (**b**) RTM; (**c**) HOSVD-RTM.

Imaging is performed with Equations (12) and (13). The image amplitude is expressed in dB. It is specified that the maximum amplitude of the sound field is 0 dB. The dynamic amplitude range is 15 dB. For the purpose of comparison, the widely used total focusing method (TFM) and reverse time migration (RTM) are also used to process the same simulation data. The imaging results are shown in Figure 6. In order to evaluate the magnitude of noise in the imaging results, the SNR is introduced in this study. The SNR [19] is defined as follows:

(14) $$SNR=20\;\log_{10}\left[\frac{\max(I_{d})}{I_{n-RMS}}\right]$$

where $I_{d}$ is the image amplitude at the point of defect and $I_{n-RMS}$ is the root mean square value of image amplitudes at other points without defect.

The image SNR of TFM, RTM, and HOSVD-RTM imaging results in Figure 6 are 14.32 dB, 22.92 dB, and 28.14 dB, respectively. TFM imaging result contains a large amount of image noise, so it is difficult to identify the center of the defect and the shape of the defect. RTM and HOSVD-RTM perform well in defect localization and focusing. The center position of the defect is determined as (69.90 mm, 23.10 mm) in RTM imaging results and (70.10 mm, 23.10 mm) in HOSVD-RTM imaging results. The noise in the RTM imaging results is reduced compared with that in TFM imaging results, but there are still more interference spots and artifacts in RTM imaging results. The SNR of HOSVD-RTM imaging results is about 14 dB larger than that in TFM imaging results and 5 dB larger than that in RTM imaging results. In addition, the defect-focusing ability of HOSVD-RTM is stronger. In HOSVD-RTM imaging results, most of the artifacts around the circular defects are eliminated and the image as a whole is almost free of noise spots, indicating the better imaging results.

In addition, the signals with different center frequencies are also used in the simulation with HOSVD-RTM. Different frequencies have little effect on their time domain waveform noise reduction effects and final imaging results, indicating that the algorithm is capable of processing ultrasonic detection signals obtained under different excitation frequencies.

#### 3.1.2. Multiple Defects

**Figure 7:**
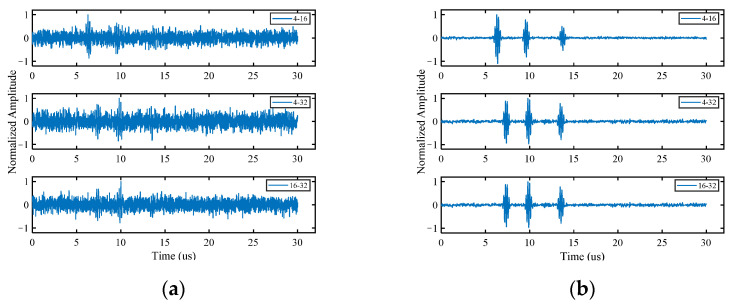
Time domain waveforms of simulated signal. (**a**) Before noise reduction; (**b**) after noise reduction.

**Figure 8:**
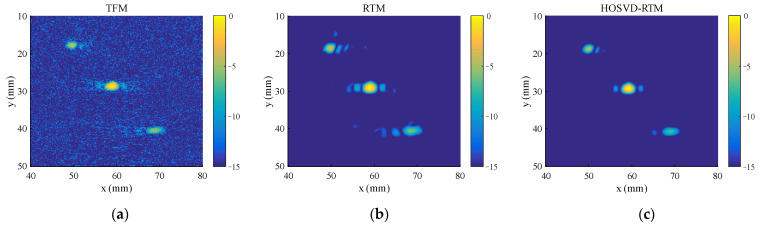
Simulated signal imaging results obtained with various algorithms. (**a**) TFM; (**b**) RTM; (**c**) HOSVD-RTM.

To test the ability of the proposed algorithm to process multiple defect signals, three circular hole defects with diameters of 2 mm (blue circles in Figure 3b) are introduced to the simulation model. The standard deviation σ of the Gaussian noise added in this section is half of the peak of the third defect echo, and the other parameters are the same as those in Section 3.1.1. The time domain waveforms before and after noise reduction are shown in Figure 7. The method still has an obvious noise reduction effect on multi-defect signals and the number of defect echoes does not affect the noise reduction effect. The final imaging results of HOSVD-RTM are compared with those of TFM and RTM, as shown in Figure 8. The HOSVD-RTM can effectively reduce the spots caused by noise and the artifacts near each defect are also reduced, proving that the HOSVD-RTM is still applicable to multiple-defect signals. A quantitative evaluation is not made in the section because the SNR is not suitable for the evaluation of imaging results with multiple defects.

#### 3.1.3. Effect of the Number of Array Elements on Imaging Results

The number of phased array elements affects the imaging effect. When the linear array has fewer elements, TFM and RTM require a high SNR signal to obtain better imaging results. In order to investigate the improvement in the imaging results under the conditions of the linear array with fewer elements, the linear arrays with 8 and 16 elements are, respectively, used to collect the FMC data in the simulation model of a single circular hole defect. Under the fixed total length of the phased array, the spacing of the elements is increased and the number of array elements is changed. Other simulation parameters remain unchanged. To evaluate the focusing effect, the lateral width of −6 dB at the defect location is calculated [39]. The image SNR and width of −6 dB for different imaging results are provided in Table 1.

**Figure 9:**
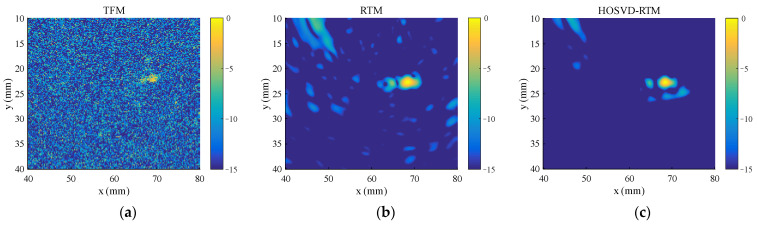
Imaging results obtained with the array composed of 8 elements and different algorithms. (**a**) TFM; (**b**) RTM; (**c**) HOSVD-RTM.

Figure 9 shows the imaging results of TFM, RTM, and HOSVD-RTM under the conditions of 8 array elements. The image SNR of TFM, RTM, and HOSVD-RTM imaging results are 12.29 dB, 17.51 dB, and 20.17 dB, respectively. HOSVD-RTM has a higher image SNR. The defects in the TFM imaging results are overlapped by a large amount of image noise and fail to be effectively highlighted, thus making it almost impossible to determine the defect location and shape. The RTM method focuses defects better than TFM method, but RTM imaging results still have more artifacts. HOSVD-RTM imaging results have fewer artifacts and more obvious defects. In terms of the lateral width of −6 dB at the defect location of the three imaging results, the defect size in HOSVD-RTM imaging result is 3.85 mm, which is 2.85 mm less than that of TFM and 0.25 mm less than that of RTM. HOSVD-RTM imaging was closer to the true size and has the better ability to focus the defect.

**Figure 10:**
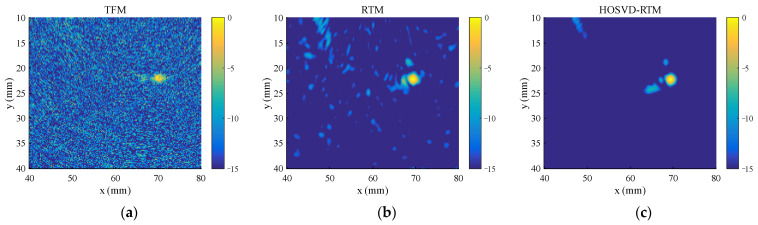
Imaging results obtained with the array composed of 16 elements and different algorithms. (**a**) TFM; (**b**) RTM; (**c**) HOSVD-RTM.

Figure 10 shows the imaging results of TFM, RTM, and HOSVD-RTM under the conditions of 16 array elements. The SNR of HOSVD-RTM imaging results is about 10 dB larger than that in TFM imaging results and 4 dB larger than that in RTM imaging results. The lateral -6dB width at the defect position is 2.13 mm, which is close to the true size of 2 mm, indicating that the focusing ability of HOSVD-RTM is also better than that of RTM and TFM.

It can be seen that HOSVD-RTM is still valid, even though the linear array has fewer elements. The imaging effect of HOSVD-RTM is significantly better than that of both RTM and TFM.

### 3.2. Experimental Verification

#### 3.2.1. One Defect in 4140 Casting Steel

The experimental object in this section is a single circular hole defect in the specimen, as indicated by the blue circle in Figure 4b. With the top left vertex as the origin, the center of the defect is located at the point (97 mm, 35 mm) and the diameter of the defect is 2 mm. The linear arrays composed of 8 and 16 elements were, respectively, used to collect the FMC data. The waves generated by the arrays were a 5-cycle Hanning-windowed tone burst with a central frequency of 10 MHz. The FMC data were collected with each element as a source to excite ultrasonic waves and all the elements in the array as receivers. The sampling frequency was 100 MHz and a time window of 5~30 μs was selected to intercept the original signal and subtract the interference of direct waves and bottom echoes.

**Figure 11:**
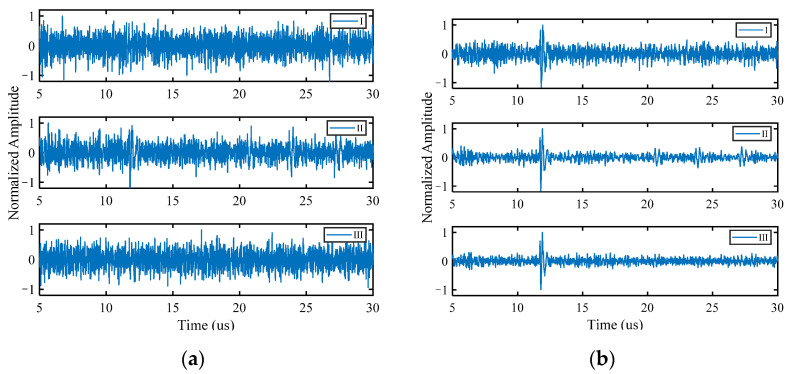
Time domain waveforms of experimental signal (**a**) before noise reduction; (**b**) after noise reduction.

Accordingly, with the same processing procedure as the simulated signal, the FMC data are subjected to Fourier transformation and then HOSVD is performed in the frequency domain. The matrix $A_{\left(1\right)}{}^{new}$ is obtained with Equation (11) and substituted into Equation (2) to display its noise reduction results. In the normalized time domain waveforms before and after noise reduction (Figure 11), “I” represents a typical signal obtained from the array composed of 8 elements; “II” and “III” represent different transmission–reception pairs’ signals obtained from the array composed of 16 elements. HOSVD-RTM method showed the good suppression effect on the noise signals obtained with two different arrays. The noise reduction ability decreased slightly when the number of arrays decreased, but the results still effectively highlighted the defect echoes, and a lot of noise was excluded.

**Figure 12:**
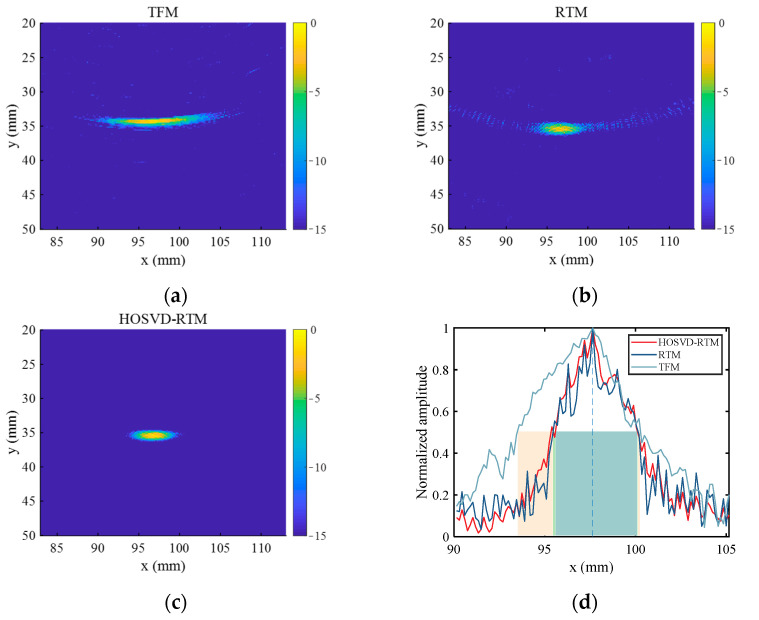
Imaging results obtained with the array composed of 8 elements and different algorithms ((**a**) TFM, (**b**) RTM, (**c**) HOSVD-RTM)); (**d**) cross-sectional view of defect location.

Figure 12a–c show the imaging results of TFM, RTM, and HOSVD-RTM, respectively, obtained with the array composed of 8 elements. The defect center was determined as the point (97.20 mm, 34.80 mm) in the TFM results, the point (97.20 mm, 35.10 mm) in the RTM results, and the point (97.00 mm, 35.10 mm) in the HOSVD-RTM results. The defect center positions in the imaging results of all three algorithms were close to the actual defect position. The image SNR is calculated with Equation (14). The corresponding image SNR values of the three imaging algorithms are 14.21 dB, 21.64 dB, and 24.09 dB, respectively. HOSVD-RTM algorithm allowed the larger image SNR, which was 69.53% larger than that of TFM algorithm and 11.32% larger than that of RTM algorithm. Figure 12d shows a partial cross-sectional view of defect location and the rectangular boxes with different colors demonstrate the lateral widths of −6 dB in the corresponding imaging results. The corresponding image SNR and lateral width of −6 dB are listed in Table 2. Similar to the simulation results, the corresponding width of −6 dB in HOSVD-RTM results was also narrower than that of TFM and RTM. The amplitude difference between the defect center and its surrounding was more obvious. The ability of HOSVD-RTM to focus the defect was much better than that of TFM and slightly better than that of RTM.

**Figure 13:**
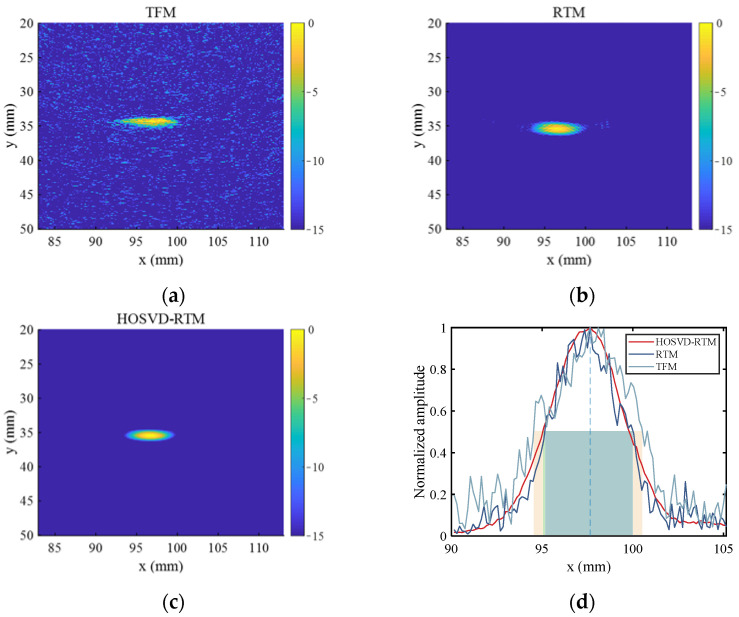
Imaging results obtained with the array composed of 16 elements and different algorithms ((**a**) TFM, (**b**) RTM, (**c**) HOSVD-RTM); (**d**) cross-sectional view of defect location.

Figure 13 shows the different imaging results and a partial cross-section at the defect location obtained with the array composed of 16 elements. Their image SNR and lateral widths of −6 dB are also listed in Table 2. HOSVD-RTM algorithm could improve the image quality, reduce the background noise, and increase the image SNR (Figure 13 and Table 2). The image SNR of HOSVD-RTM method was 67.39% larger than that of the TFM algorithm. The image SNR of HOSVD-RTM method was 14.01% larger than that of the RTM algorithm.

#### 3.2.2. Multiple Defects in 4140 Casting Steel

To further verify the performance of the proposed algorithm in processing multiple defect signals, in this section, two circular hole defects in the specimen were selected as the experimental detection objects (gray circles in Figure 4b). The defect diameter was 4 mm, and the defect centers were, respectively, located at the points (261 mm, 102 mm) and (257 mm, 108 mm). The linear arrays composed of 8 and 16 elements were, respectively, used to acquire the FMC data and the wave generated by the array was a 5-cycle Hanning-windowed tone burst with a central frequency of 5 MHz. The sampling frequency was 100 MHz and a time window of 20–60 μs was selected to intercept the original signal and subtract the interferences of direct waves and bottom echoes.

**Figure 14:**
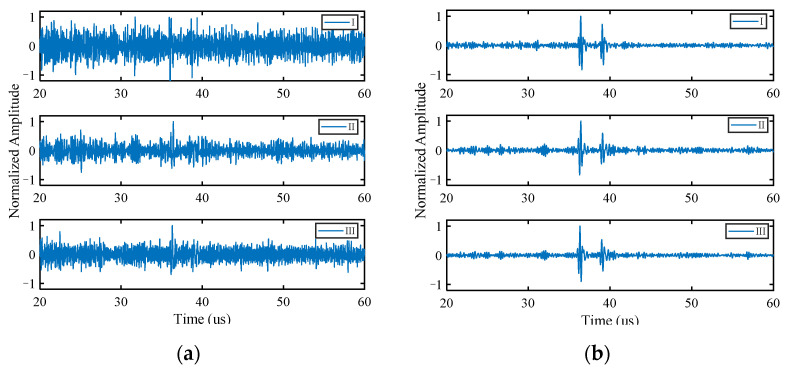
Time domain waveforms of experimental signal. (**a**) Before noise reduction; (**b**) after noise reduction.

In the normalized time domain waveforms before and after noise reduction (Figure 14), “I” represents a typical signal obtained from the array composed of 8 elements; “II” and “III” represent different transmission–reception pairs’ signals obtained from the array composed of 16 elements. The time domain waveforms showed that multiple defect echoes did not affect the noise reduction ability of the method, and HOSVD-RTM can still highlight two defect echoes and reduce a lot of noise, as indicated in the simulation results.

**Figure 15:**
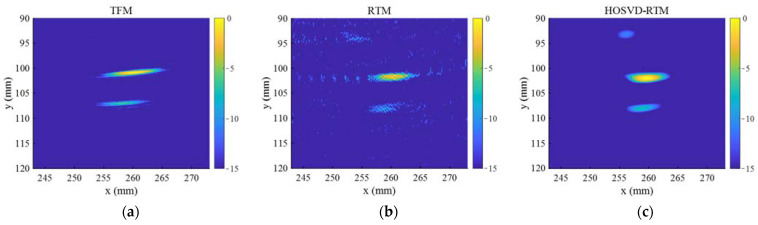
Imaging results obtained with the array composed of 8 elements and 3 algorithms: (**a**) TFM, (**b**) RTM, and (**c**) HOSVD-RTM.

**Figure 16:**
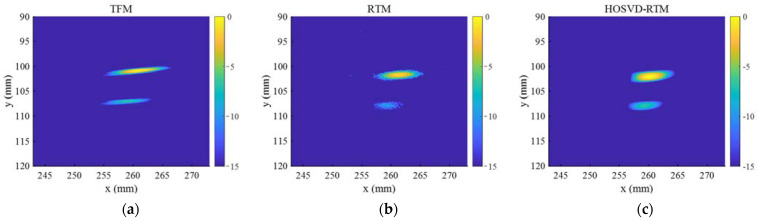
Imaging results obtained with the array composed of 16 elements and 3 algorithms: (**a**) TFM, (**b**) RTM, and (**c**) HOSVD-RTM.

Figure 15 shows the imaging results of three imaging methods (TFM, RTM, and HOSVD-RTM) obtained with the array composed of 8 elements. Figure 16 shows the imaging results of three imaging methods obtained with the array composed of 16 elements. The centers of the defects in imaging results of all three algorithms were close to their actual positions, indicating the higher accuracy. The second defect was more obvious and more easily observed in the HOSVD-RTM imaging results than that in the TFM and RTM imaging results. In the TFM and RTM imaging results, the echo signal of the second defect was not prominent enough and overlapped by a lot of noise signals. Table 3 lists the lateral widths of −6 dB of the two defects in different imaging results. The TFM image had the widest width of the two defects and the RTM and HOSVD-RTM images had the similar widths of the two defects. Similar to the individual defect imaging results, HOSVD-RTM focused the defects significantly better than TFM and slightly better than RTM.

## 4. Conclusions

In this study, an ultrasonic RTM imaging method based on HOSVD was developed and applied successfully to detect defects in the materials with high noise levels. In this method, we firstly constructed the three-dimensional full-matrix data into a third-order tensor and then performed HOSVD. Finally, we used the processed data for RTM imaging. This approach removes noise from all signals in a single step and avoids the DORT algorithm’s difficulty in determining the number of singular values. Numerical simulations and experimental tests were conducted to demonstrate the effectiveness of this method. The results show that the method is suitable for a variety of situations, such as detection of multiple defects, excitation signals of different frequencies, and phased arrays with different numbers of elements.

The SNR of the imaging results of this algorithm is compared with conventional TFM and RTM imaging algorithms. The imaging results demonstrate that HOSVD-RTM has a larger image SNR and can effectively reduce spots caused by noise and artifacts around defects. Compared with TFM and RTM, this method increases the SNR of the imaging results by approximately 12 dB and 4 dB, respectively. The lateral width of −6 dB at the defect location in the imaging results is further investigated and HOSVD-RTM focuses defects much better than TFM and slightly better than RTM. The noise reduction effect in the A-scan data is significantly better than that in the imaging results because TFM and RTM imaging results are the superimposing consequence of multiple array elements. In addition, the application of the tensor analysis method in array signal data processing and information extraction still needs to be further explored. The proposed method is applicable to the ultrasound test and other fields, such as geological exploration and bioimaging.

## Figures and Tables

**Table 1:** Image SNR and width of −6 dB in different imaging results.

Measurement Content	Number of Array Elements	TFM	RTM	HOSVD-RTM
Image SNR (dB)	8	12.29	17.51	20.17
	16	13.22	19.79	23.83
Width of −6 dB (mm)	8	6.69	4.10	3.85
	16	3.76	2.22	2.13

**Table 2:** Image SNR and lateral width of −6 dB of different imaging results.

Measurement Content	Number of Array Elements	TFM	RTM	HOSVD-RTM
Image SNR (dB)	8	14.21	21.64	24.09
	16	16.53	24.27	27.67
Width of −6 dB (mm)	8	6.32	4.63	4.61
	16	5.63	4.57	4.57

**Table 3:** Lateral width of −6 dB for the defects in different imaging results (mm).

Array Type	TFM		RTM		HORTM	
	Defect 1	Defect 2	Defect 1	Defect 2	Defect 1	Defect 2
Array composed of 8 elements	6.8	8.6	5.6	7.0	5.8	6.3
Array composed of 16 elements	6.9	8.0	5.6	6.0	5.7	6.2
